# Evaluation of HIV infection in febrile patients visiting health centers in Lagos, Nigeria

**DOI:** 10.1186/s13104-022-05961-0

**Published:** 2022-02-19

**Authors:** Ololade O. Akinnusi, Adebayo J. Bello, Isaac A. Adeleye, Jerry John Nutor

**Affiliations:** 1 Department of Microbiology, University of Lagos, Akoka, Lagos State Nigeria; 2 Department of Cell Biology and Genetics, University of Lagos, Akoka, Lagos State Nigeria; 3 Evercare Laboratory, Evercare Hospital, Lekki, Lagos State Nigeria; 4 Department of Family Health Care Nursing, School of Nursing, University of California San Francisco, 2 Koret Way, Suite N431G, San Francisco, CA 94143 USA

**Keywords:** Malaria, Sub-Sahara Africa, Prevalence, Hospital

## Abstract

**Objective:**

Acute febrile infections compatible with malaria are the most prevalent presentation at sub-Saharan African health clinics, accounting for 30–50% of outpatient visits. Acute human immunodeficiency virus (HIV) infection can mimic acute malaria symptoms. As a result, screening people with malaria symptoms for HIV infection is critical. The goal of our study was to find out how common HIV infection was among feverish patients.

**Results:**

Out of the 310 individuals screened, 9 (3.0%) had HIV-1 infection, with 5 (55.5%) being females and 4 (44.4%) being males. This study found no evidence of HIV-2 infection or HIV-1/HIV-2 co-infection. HIV infection was found in 1–3% of patients with probable malaria at different sites in Lagos, Nigeria.

## Introduction

The human immunodeficiency virus (HIV) epidemic continues to be a problem globally and is a severe public health concern in developing countries, including Nigeria. The investigations conducted by Olaleye and colleagues and Zeh and colleagues showed HIV-1 to be more prevalent in Nigeria than HIV-2 [1, 2].

Nigeria has the world’s second-largest HIV pandemic [3]. Although the prevalence of HIV among adults in Nigeria is significantly lower (1.3%) than in other Sub-Saharan African nations such as South Africa (19%) and Zambia (11.5%), the country’s population implies that 1.8 million individuals were infected in 2019 [4]. Better surveillance has been credited with recent decreases in prevalence estimates for the country [5].

Nonetheless, the Joint United Nations Programme on HIV and AIDS (UNAIDS) estimates that Nigeria accounted for around two-thirds of new HIV infections in West and Central Africa in 2019. Every year, the country, together with South Africa and Uganda, accounts for almost half of all new HIV infections in Sub-Saharan Africa [4]. This is despite achieving a 13% drop in new infections between 2010 and 2019 [4].

In Nigeria, 80% of new HIV infections are caused by unprotected heterosexual intercourse, with the bulk of remaining HIV infections happening in critically impacted communities such as sex workers [3].

Co-infection with HIV-1 and HIV-2 occurs in countries where both viruses are in circulation. Although progression, as implicated by higher viral loads, is driven by HIV-1 in most dually infected individuals [6], this is not always the case [7].

Most African adults who acquire HIV-1 are frequently treated for malaria without evaluation for sexually transmitted infections (STIs) or HIV risk [8]. It is essential that health care professionals consider HIV infection as a potential cause of febrile illnesses. In addition, individuals with early HIV-1 infection who seek healthcare for symptoms prior to seroconversion are highly contagious and may be responsible for a large number of new HIV-1 infections [9]. Though some individuals at this stage of the infection remain without symptoms, a few of them experience an acute ‘malaria-like’ illness approximately two weeks after infection [8, 10].

Subsequently, it has been deduced that the diagnosis of patients presenting with fever is majorly focused on malaria [11], and guidelines are not well defined in terms of evaluating aetiologies other than malaria and the current practice in Nigeria shows very poor adoption of HIV-1 testing in this category of patients because it is not currently considered in the diagnosis. To this end, this study was conducted to ascertain the burden of HIV infection among febrile patients, although, a subset of this study was presented and published as a conference abstract [12].

## Main text

### Methodology

#### Study centre

The study was conducted in a non-governmental HIV/AIDS clinic, acquired immune deficiency syndrome (AIDS) Prevention Initiative in Nigeria (APIN) clinic situated in the University of Lagos Teaching Hospital (LUTH). The clinic is a referral center for counseling, testing, and providing care for HIV patients and has a yearly patients enrolment of over 1000 (it is equipped with a standard virology laboratory; hence, the choice as a study center where the samples collected were tested for HIV).

#### Study population

The study population consisted of patients with fever of unknown origin associated with 39–41 °C temperature between ages 1 to 84 years who visited the selected health care centers in Lagos State.

Study participants were 310 individuals who sought health care for febrile illness at General Hospitals (Orile-Agege, Shomolu, Lagos Island, and Surulere) in Lagos State. The minimum sample size calculated the study population (N), determined using the equation as described by Pourhoseingholi et al. in 2013 [21]: n = Z^2^P(1−P)/d^2^ = 48.

Where n = sample size,

Z = statistics for a level of 95% confidence interval = 1.96,

d = precision (allowable error) = 5% = 0.05.

P = prevalence rate (3.17%) as reported by NACA [13, 14].

The population of Orile-Agege, Shomolu, Lagos Island, and Surulere are reported as 635,900, 555,800, 292,900 and 692,500 persons respectively [15]. Meanwhile, a minimum of 48 participants were calculated for the study, but was marked up to 310 for improved quality of data.

#### Recruitment of study subjects

Participants were recruited from Lagos Island General Hospital, Shomolu Health Center, Orile Agege General Hospital, and Randle General Hospital, Surulere. The acceptance rate was 100%; all eligible participants approached by the research assistants agreed to participate in the study. The attending nurse approached patients who met the inclusion criteria and briefly explained the study to them. After receiving consent from the participants, the nurse introduced the participants to trained research assistants who explained the study comprehensively to them and took their signed consent form and initial biographical details to enroll them in the study. Participants were administered a standardized questionnaire after obtaining their informed consent. The patient samples were afterward transported to the APIN clinic for analysis.

#### Inclusion/exclusion criteria

All patients who presented with fever of unknown origin with temperature between 39° and 41 °C and were screened malaria negative in the selected hospitals were eligible for the study. Patients who were malaria positive during screening or patients with a known medical history of other viral infections or terminal diseases were excluded.

#### Specimen collection and laboratory analysis

Five milliliters of venous blood were aseptically drawn into the plain vacutainer bottle and was allowed to stand undisturbed for 2 h, after which it was spinned in the centrifuge at 15,000 rpm for 5 min. The resulting sera were aseptically transferred into another plain vacutainer bottle, properly labelled, and kept at 2–8 °C for the serological assay.

#### Laboratory procedures for the immunochromatographic tests

Serum samples were analyzed using three qualitative tests: Alere^Tm^ HIV 1 and 2, Determine^Tm^ kits (Alere Medical Co., Ltd, Chiba, Japan) to detect all the positive samples. Presumptive positive samples were further tested using Uni-Gold^Tm^ HIV kits (Trinity Biotech PLC, Co Wicklow, Ireland). Thereafter Standard Diagnostics BIOLINE HIV-1/2 3.0 was used to identify HIV-1, HIV-2, and HIV-1/HIV-2 co-infection. All the analyses were carried out following strictly the manufacturer’s instructions.

#### Statistical analysis

The Statistical Package for the Social Sciences (SPSS) version 10.0, Chicago, IL, USA was used to calculate mean values and percentages.

#### Ethical approval

The study was conducted following the Declaration of Helsinki for human subjects research. The protocol for this study was approved by the Health Research Ethics Committee, College of Medicine of the University of Lagos, with reference number CMUL/HREC/06/17/188. Participants consented before enrolment using consent forms approved by the Ethics and Research Committee of the LUTH. Interested participants aged 18 enrolled in the study after signing a written informed consent form. No minor was recruited without informed parental consent. In addition to informed parental consent, informed written assent was obtained from all children below 18 years of age prior to their participation.

### Results

The sample's characteristics are shown in Table 1. The study population included 310 individuals aged 1–84 years with a fever of unknown cause and 39–41 °C temperature who visited hospitals or health care centers/facilities in specified geographical locations of Lagos state.

**Table 1:** Socio-demographic characteristics of participants

Variables	Character	Number of participants (%)
Marital status	Single	161 (51.9)
	Married	140 (45.2)
	Divorced	6 (1.9)
	Widowed	3 (1.0)
Religion	Christianity	146 (47.0)
	Islam	155 (50.0)
	Traditional	3 (1.0)
	Others	6 (2.0)
Tribe	Yoruba	211 (68.1)
	Igbo	60 (19.4)
	Hausa	26 (8.4)
	Others	13 (4.2)
Educational level	Primary	81 (26.1)
	Secondary	132 (42.6)
	Tertiary	80 (25.8)
	None	17 (5.5)
Occupation	Civil servant	50 (16.1)
	Entrepreneur	53 (17.1)
	Artisan	146 (47.1)
	Farmer	7 (2.3)
	Unemployed	54 (17.4)

The health centers visited for this study were; Lagos Island general hospital (74 patients; 28 males and 46 females), Shomolu health center (49 patients; 16 males and 33 females), Orile Agege general hospital (97 patients; 31 males and 66 females) and Randle general hospital, Surulere (90 patients; 33 males and 57 females).

Early HIV infection was diagnosed in 9 (3.0%) of the 310 individuals enrolled, with 5 (55.5%) females and 4 (44.4%) males. The nine positive patients were all HIV-1, no HIV-2, and no HIV-1/HIV-2.

Table 2 illustrates a breakdown of all positive samples, demonstrating that there are nine patients, five of whom are females and four of whom are males.

**Table 2:** Gender and age distribution of HIV-1 infection among the cohort

	Sample	Determine	Unigold	SD-Bioline
Gender distribution of HIV infections				
Male	Negative	104	107	104
	Positive	4	1	–
	HIV-1	–	–	4
	Total	108	108	108
	% Positive	3.70	0.93	3.70
Female	Negative	197	198	197
	Positive	5	4	–
	HIV-1	–	–	5
	Total	202	202	202
	% Positive	2.48	1.98	2.48

Age group	Positive	Negative	Total	% Infection
HIV age distribution among the cohort				
< 1–15	1	67	68	1.47
16–45	6	159	164	3.66
46 and above	2	75	78	2.56
Total	9	301	310	2.9

HIV-1 seropositive results and location						
			Rapid HIV test result			
S/N	Sex	Age range (years)	Determine	Unigold	SD-Bioline	Location
A03	F	40–44	P	P	HIV-1	Island Gen. Hosp.
D13	F	45–49	P	P	HIV-1	Orile-Agege
F06	F	45–49	P	P	HIV-1	Randle Gen. Hosp.
F15	F	50–54	P	N	HIV-1	Orile-Agege
F20	F	30–34	P	P	HIV-1	Orile-Agege
G07	M	15–19	P	P	HIV-1	Island Gen. Hosp.
H06	M	25–29	P	N	HIV-1	Island Gen. Hosp.
J03	M	30–34	P	N	HIV-1	Orile-Agege
J07	M	0–4	P	N	HIV-1	Orile-Agege

*P* Positive, *N* Negative

Furthermore, the two rapid kits used in this study had varying sensitivity, with Alere^Tm^ HIV 1 and 2 Determine^Tm^ showing greater sensitivity for the HIV antigen than Uni-Gold^Tm^. The third kit, SD-Bioline, served as a tiebreaker and a differentiating kit, comprehensively differentiating the positive samples into the HIV-1 type.

Additionally, a higher proportion (5/9) of the positive persons (55.5%) are patients of the Orile Agege General Hospital, which is located in a rural part of Lagos, Nigeria.

The gender prevalence rate among male and female feverish patients from the samples evaluated shows a greater prevalence rate in male (3.70%) compared to female (2.48%) febrile patients.

Table 2 indicates the HIV prevalence rate among various age groups, with a mean prevalence rate of 3.0%. The infection was more prevalent among individuals aged 16–45, with a 3.66% HIV prevalence (Table 2); the mean age was 32.17 years.

## Discussion

The use of the specified rapid kits, Alere^Tm^ HIV 1 and 2 Determine^Tm^ and SD BIOLINE HIV-1/2 3.0 for diagnosis in this study is consistent with Nsagha et al*.,* 2012, who studied HIV-1/HIV-2 co-infection among volunteer counseling and testing individuals at a regional health center in Cameroon.

These rapid tests take advantage of p24, a unique protein that is found in the HIV viral core or coat protein. This protein is found in high concentrations in the blood of newly infected individuals during the brief period between initial infection and seroconversion, making the p24 antigen valuable in identifying early HIV status.

This study used an additional kit (Uni-Gold™ Recombigen® HIV) to follow the national protocol established by the Nigeria Federal Ministry of Health, which calls for the use of three rapid tests, with the third serving as a tiebreaker in the event of an indeterminate result.

The prevalence estimate in this study of 3.0% is consistent with prior research that indicated acute HIV infection to be a cause of fever in adult patients in Sub-Saharan Africa [8, 16]. In Mozambique, 3.3% of HIV-seronegative people who went to a district hospital with fever were discovered to have acute HIV infection [13]. Another research carried out in coastal Kenya in 2011 discovered that many individuals sought quick medical assistance after contracting HIV and were often given antimalarial drugs [8].

The age groups distribution revealed that the adult age group (16–45 years) had the greatest HIV prevalence rate, while the pediatric age group had the lowest incidence. This trend is consistent with most reports, which designate sexually active adults as the most vulnerable age group for HIV infection [17, 18]. UNAIDS has previously reported that the HIV prevalence in Nigeria is higher among people between the ages of 15 and 49 [4]. Abah's research has also shown that the age bracket with the greatest HIV prevalence in Nigeria's southwest region is 30–39 [19]. Factors such as alcoholism, unprotected casual intercourse, and job hazards, particularly among migrant workers, have also been identified as major HIV transmission drivers, accounting for high infection rates among adults of reproductive age, according to health promotion experts.

This study found a greater prevalence (3.7%) in the male population compared to the female population (2.48%), which contradicts the findings of Bashorun and colleagues [20], who estimated a higher prevalence of HIV in the female population. We found no patients with HIV-2, which contrasts with the findings of a study conducted in Mali [21] that found more HIV-2 than HIV-1, as well as a similar study conducted in Cameroon by Nsagha and colleagues [22], which reported 79.5% HIV-1, 1.3% HIV-2, and 19.2% HIV-1/ HIV-2 co-infection. The dramatic fall in HIV-2 and HIV-1/HIV-2 co-infection in this study shows that the co-infection rate may decrease and that HIV-2 is probably tending towards extinction in Nigeria.

Failure to detect or treat HIV infection in pregnant women increases the risk of transmission to the child during delivery, which is greatly increased with early HIV infection, as demonstrated by the one-year-old HIV-positive male patient (Table 1).

With an MTCT (mother-to-child transmission) rate of over 30% and approximately 75,000 new HIV infections per year, Nigeria accounts for roughly 30% of the overall prevalence of MTCT worldwide [23]. In a study conducted by Ezeanolue and colleagues, only 46,200 HIV-infected pregnant women received ARV out of an expected 210 000, resulting in a coverage rate of only 22% [24]].

## Conclusion

This study demonstrates that by focusing on individuals experiencing febrile diseases, it is possible to identify many Nigerians with early HIV infection. The identification of early HIV infections enables the tracking of HIV epidemiological data, which can be used to evaluate the efficacy of current diagnostic, treatment, and prevention strategies and also to drive essential policy adjustments. Furthermore, HIV patients should be looked after and administered antiretroviral therapy following established recommendations.

## Limitations

The entire study was based on rapid test kits, suggesting why HIV-2 infection was not detected in the samples tested. We believe that molecular testing with probes and primers specific for HIV-2 could be more helpful in detecting HIV-1 and 2 co-infection in feverish patients.

## Data Availability

Raw data still available with O.O.A, he should be contacted to gain access to data not presented in this manuscript.

## Abbreviations

AIDS: Acquired immune deficiency syndrome
APIN: AIDS Prevention Initiative in Nigeria
FMH: Federal Ministry of Health
HIV: Human Immunodeficiency Virus
LUTH: Lagos University Teaching Hospital
MTCT: Mother to Child Transmission
SPSS: Statistical Package for the Social Sciences
STI: Sexually Transmitted Infection
UNAIDS: Joint United Nations Programme on HIV and AIDS
